# AI-Derived Carpal Compactness Metrics as Quantitative Imaging Measures of Global Carpal Architecture in Early Rheumatoid Arthritis

**DOI:** 10.3390/jimaging12080363

**Published:** 2026-08-08

**Authors:** Jiajing Zhou, Haolin Wang, Ikuma Nakagawa, Shun Tanimura, Yuhei Shibata, Ken Nagahata, Tamotsu Kamishima

**Affiliations:** 1 Faculty of Health Sciences, Hokkaido University, Sapporo 060-0812, Hokkaido, Japan; zzzjjj99@163.com (J.Z.); ktamotamo2@hs.hokudai.ac.jp (T.K.); 2 Graduate School of Health Sciences, Hokkaido University, Sapporo 060-0812, Hokkaido, Japan; 3 Hokkaido Medical Center for Rheumatic Diseases, Sapporo 063-0811, Hokkaido, Japan; ikuma.nakagawa@gmail.com (I.N.); tanitani1016@gmail.com (S.T.); uhey48ta@gmail.com (Y.S.); nagahataken@gmail.com (K.N.)

**Keywords:** rheumatoid arthritis, artificial intelligence, quantitative imaging measures, wrist radiography, carpal spatial architecture

## Abstract

Rheumatoid arthritis (RA) frequently involves the wrist joints, where early inflammation may induce subtle changes in carpal spatial architecture. This study investigated whether baseline (BL) ultrasound inflammation is associated with longitudinal changes in AI-derived compactness metrics and whether these metrics provide information complementary to conventional wrist radiographic scores. This study included 159 patients with early RA who underwent BL and follow-up (FU) bilateral wrist radiography and BL wrist ultrasound. Four AI-derived centroid-based compactness metrics—root-mean-square-radius (RMSR), mean pairwise distance (MPD), median radius (R50), and 90th-percentile-radius (R90)—were calculated, and their annualized changes were evaluated in relation to conventional wrist SvdH scores and BL ultrasound inflammation, including gray-scale (GS), power Doppler (PD), GS+PD, and vascularity percentage (VS%). Associations were assessed using correlation and subgroup analyses. AI-derived compactness metrics at BL and FU, as well as their annualized changes, were not significantly associated with conventional wrist radiographic scores. In contrast, BL ultrasound inflammation was significantly associated with subsequent increases in compactness metrics. BL GS, PD, and GS+PD scores showed significant positive correlations with annualized changes in RMSR, MPD, R50, and R90 (r = 0.18–0.25, *p* < 0.05). Subgroup analyses similarly demonstrated greater increases in compactness metrics in patients with positive GS or PD activity. Annualized changes in AI-derived compactness metrics were associated with baseline ultrasound inflammation but not with changes in conventional wrist radiographic scores. These findings suggest that AI-derived compactness metrics may provide complementary quantitative information on global carpal spatial architecture in early RA.

## 1. Introduction

Rheumatoid arthritis (RA) is a chronic autoimmune disease characterized by synovial inflammation and progressive joint damage [1]. It predominantly affects small joints and their surrounding soft tissues, leading to pain, joint deformity, functional impairment, reduced quality of life, and a substantial burden on patients and society [2]. The wrist is one of the earliest and most frequently affected anatomical sites in RA [3]. Because the wrist plays an important role in hand function, early inflammation and structural changes in this region may contribute to restricted motion and long-term disability [4]. In the early phase of RA, inflammatory activity may already induce subtle changes in carpal spatial architecture that are not fully reflected by conventional imaging scores. Therefore, sensitive assessment of wrist structural changes in early RA is important for understanding inflammation-associated disease progression.

Conventional radiography remains one of the most widely used imaging modalities for assessing structural damage in RA because of its low cost, broad availability, and suitability for long-term follow-up [5]. Conventional radiographic scoring methods, including the Sharp/van der Heijde (SvdH) scoring system, are widely used to evaluate bone erosion (BE) and joint space narrowing (JSN) and provide information on cumulative structural damage and radiographic progression [6,7]. However, these scoring methods primarily assess established radiographic damage and are semi-quantitative [8]. In the wrist, visual assessment is particularly challenging because multiple small carpal bones have complex morphology, close spatial relationships, and substantial overlap on two-dimensional radiographs [9]. Consequently, conventional wrist radiographic scores may be insufficiently sensitive to early, subtle, or continuous changes in carpal spatial architecture. Thus, radiographic assessment based on local structural damage may not fully reflect inflammation-associated carpal changes in early RA.

Musculoskeletal ultrasound provides important complementary information on synovial inflammation in RA [10]. Gray-scale ultrasound (GS) is commonly scored semi-quantitatively and is used to assess synovitis-related structural changes, such as synovial hypertrophy and joint effusion [11,12]. Power Doppler ultrasound (PD) detects intra-synovial blood flow signals and reflects synovial vascularity and active inflammation [11,13]. Vascularity percentage (VS%), derived from Doppler signals, can further provide quantitative information on the degree of synovial vascularity [14]. Previous studies on ultrasound inflammation have commonly used conventional radiographic progression, BE, or JSN as structural outcomes [15]. However, it remains unclear whether baseline (BL) ultrasound inflammation is associated with longitudinal changes in global carpal spatial architecture.

Recent advances in artificial intelligence-based image analysis have enabled automated segmentation of wrist bones on radiographs [16]. Automated segmentation frameworks, such as the adversarial-prior-based dual-path merging network (AP-DPM), enable extraction of individual carpal bone masks and centroid coordinates from standard wrist radiographs [17]. The performance of wrist bone instance segmentation has also recently been evaluated more comprehensively in the multi-institutional RAM-W600 benchmark [18]. Based on the resulting segmentation masks, centroid coordinates can be extracted and used to derive compactness metrics that characterize the global spatial organization of the carpus [19]. In this study, we focused on four predefined centroid-based compactness metrics: root-mean-square-radius (RMSR), mean-pairwise-distance (MPD), median-radius (R50), and 90th-percentile-radius (R90). RMSR and MPD quantify overall centroid dispersion and inter-bone spacing, whereas R50 and R90 provide robust measures of the radial distribution of the carpal centroids. Together, these complementary metrics provide a quantitative description of overall carpal geometry from different geometric perspectives and may facilitate the detection of subtle architectural alterations associated with early inflammatory changes in RA.

However, the significance of AI-derived carpal compactness metrics as quantitative imaging measures in early RA has not been fully elucidated. Conventional wrist radiographic scores mainly assess local JSN at predefined wrist joints and reflect established radiographic structural damage [7]. In contrast, centroid-based compactness metrics describe the carpus’s global spatial configuration. Therefore, these metrics may provide complementary information beyond conventional radiographic scoring. Nevertheless, their relationship with inflammatory activity and their potential biological interpretation remain unclear.

To address these gaps, this study evaluated AI-derived centroid-based compactness metrics as potential quantitative imaging measures of global carpal spatial architecture in patients with early RA. We examined their relationships with conventional wrist radiographic scores and BL ultrasound inflammatory activity, and further assessed whether BL ultrasound inflammation was associated with subsequent annualized changes in these metrics.

## 2. Materials and Methods

### 2.1. Patients

The institutional database initially contained 248 patients with RA who had available wrist radiographs and relevant clinical imaging data. Because this study focused on early RA, patients with a disease duration greater than 2 years were excluded. Patients were also excluded if BL or FU wrist radiographs were unavailable or if radiographs were unsuitable for AI-based carpal analysis. Before quantitative analysis, all radiographs underwent image quality assessment to determine their suitability for the centroid-based compactness analysis framework. Radiographs showing severe carpal destruction with loss of normal carpal bone contours, extensive bony ankylosis involving multiple carpal bones, or advanced erosive changes resulting in indistinguishable bone boundaries were excluded because reliable segmentation and centroid extraction of individual carpal bones could not be achieved. In such cases, compactness-related metrics could not be calculated accurately.

After applying the eligibility and image quality criteria, 159 patients were included in the final analysis. All included patients had a disease duration of ≤2 years and underwent bilateral posteroanterior (PA) wrist radiography at both BL and FU. BL bilateral wrist ultrasound data were also available for all included patients.

**Figure 1:**
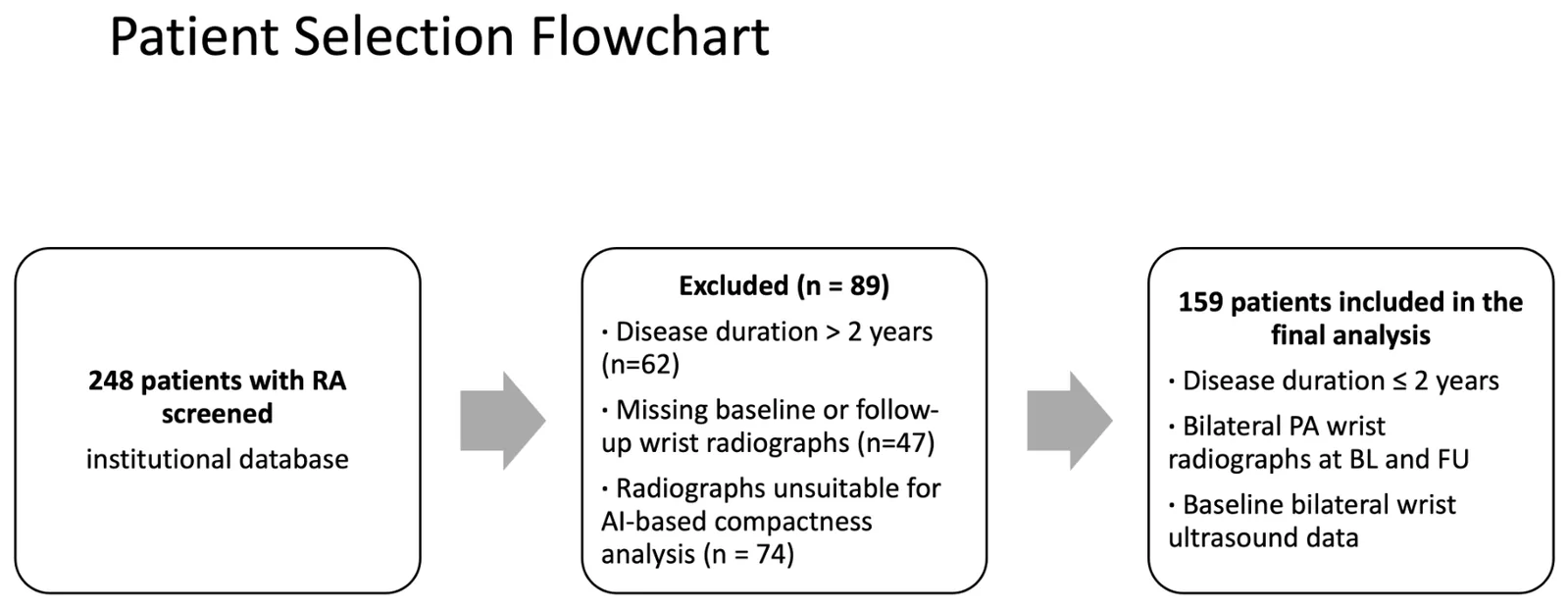
Patient selection flowchart. The institutional database initially contained 248 patients with rheumatoid arthritis. After exclusion of patients with disease duration >2 years, missing imaging or ultrasound data, and cases unsuitable for centroid-based compactness analysis, 159 patients were included in the final analysis. Some patients fulfilled more than one exclusion criterion; therefore, the numbers for individual exclusion criteria are not mutually exclusive and do not sum to the total number of excluded patients.

The patient selection process is shown in Figure 1. The demographic, clinical, ultrasound, radiographic, and AI-derived compactness metric characteristics of the study cohort are summarized in Table 1.

### 2.2. Radiographic Assessment

All digital hand radiographs were acquired using the same imaging system at a single center, ensuring a consistent spatial resolution with a pixel size of 0.15 mm × 0.15 mm. Although the centroid-based compactness metrics and their annualized changes are reported in pixels and pixels/year, they can be directly converted to physical dimensions using this constant scaling factor (1 pixel = 0.15 mm). For reference, annual changes of approximately 2–4 pixels correspond to physical changes of approximately 0.3–0.6 mm.

Conventional wrist radiographic damage was assessed using a wrist-specific modification of the SvdH JSN scoring system [7]. All wrist radiographs were independently reviewed by an experienced radiologist. JSN was assessed in six wrist joint regions, including the radiocarpal (RC), scaphotrapeziotrapezoid (STT), scaphocapitate (SC), third carpometacarpal (CM3), fourth carpometacarpal (CM4), and fifth carpometacarpal (CM5) joints.

Each joint was graded on a five-point ordinal scale ranging from 0 to 4, where 0 indicated a normal joint space, 1 represented focal narrowing or questionable change, 2 indicated generalized narrowing with more than 50% of the original joint space remaining, 3 indicated generalized narrowing with less than 50% of the original joint space remaining or subluxation, and 4 represented complete joint space loss, bony ankylosis, or joint dislocation.

Individual joint scores were summed to generate a total wrist radiographic score for each patient at BL and FU. These scores were subsequently used to evaluate their relationships with AI-derived compactness metrics.

### 2.3. Ultrasound Assessment

BL wrist ultrasound examinations were used to assess inflammatory activity. Ultrasound inflammatory metrics included GS, PD, the combined GS+PD score, and VS% [20].

Each ultrasound examination was performed and scored by one of two experienced sonographers as part of routine clinical practice, and the final ultrasound scores recorded in the clinical database were used for the present analysis. The ultrasound acquisition and assessment procedures followed the same standardized methodology and observer training framework used in our previous investigations [21,22]. In a previous study using this methodology, excellent inter-observer agreement was demonstrated for both conventional semiquantitative scoring and quantitative vascularity assessment, with intraclass correlation coefficients of 0.982 for both methods [21]. Total wrist GS and PD scores were obtained from the ultrasound database. The combined GS+PD score was calculated as the sum of the GS and PD scores, whereas VS% was used as a quantitative measure of Doppler signal activity and synovial vascularity.

BL GS, PD, GS+PD, and VS% were used to investigate correlations between BL ultrasound inflammatory metrics and BL AI-derived compactness metrics, as well as correlations between BL ultrasound inflammatory metrics and subsequent annualized changes in AI-derived compactness metrics.

### 2.4. AI-Derived Compactness Metrics

AI-derived compactness metrics were calculated from wrist radiographs using the AP-DPM segmentation model [17]. AP-DPM is an anatomical-prior-guided segmentation framework designed for wrist radiographs, in which multiple carpal bones frequently overlap on projection images. The model employs a dual-path architecture that separately predicts the global carpal bone regions and anatomically overlapping areas. These outputs are subsequently integrated through a lightweight merging module to improve the delineation of blurred bone boundaries and narrow intercarpal spaces. In addition, an adversarial discriminator is incorporated to impose anatomical plausibility on the predicted masks by penalizing structurally implausible bone shapes. A two-stage training strategy was used to stabilize model optimization and to balance pixel-level segmentation accuracy with anatomical consistency. The AP-DPM model was previously validated for automated wrist bone segmentation and achieved a Dice similarity coefficient of approximately 0.98 in its original validation study [17]. More recently, wrist bone instance segmentation performance was comprehensively evaluated in the RAM-W600 benchmark using multiple complementary metrics, including the Dice similarity coefficient (DSC), normalized surface Dice (NSD), volumetric overlap error (VOE), and mean surface distance (MSD) [18]. Across the supervised models evaluated in RAM-W600, the best reported results reached a DSC of 97.75%, an NSD of 90.71%, a VOE of 4.35%, and an MSD of 1.05 pixels. These metrics provide complementary assessments of mask overlap, boundary agreement, volumetric discrepancy, and surface distance, respectively. The same pretrained AP-DPM model was directly applied in the present study without further modification.

Using the segmentation masks generated by AP-DPM, the two-dimensional centroid of each segmented carpal bone was extracted, and the centroid-based compactness metrics were calculated as previously described [19]. Let $c_{k}$ denote the two-dimensional centroid coordinate of the *k*-th segmented carpal bone (k=1,…,n), where *n* represents the number of segmented carpal bones. The global centroid was defined as

(1) $$\mu =\frac{1}{n}\sum_{k=1}^{n}c_{k}.$$

**Figure 2:**
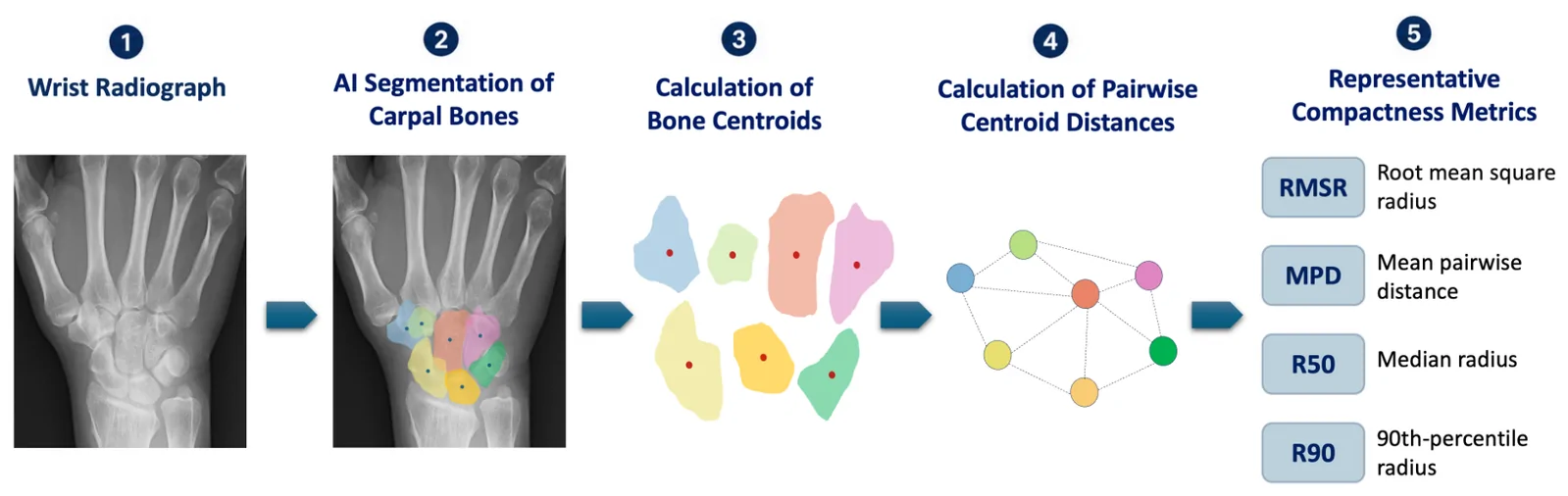
Overview of the AI-based compactness analysis workflow. Wrist radiographs were processed using an automated carpal bone segmentation framework to obtain individual carpal bone masks. Bone centroid coordinates were extracted from the segmented carpal bones, and pairwise centroid distances were calculated. Four representative compactness metrics were subsequently derived: RMSR (root-mean-square-radius), MPD (mean-pairwise-distance), R50 (median-radius), and R90 (90th-percentile-radius).

Pairwise Euclidean distances between carpal centroids and radial distances from each centroid to the global centroid were then calculated. The overall workflow of the AI-based compactness analysis is illustrated in Figure 2.

Four predefined centroid-based compactness metrics were derived: RMSR, MPD, R50 and R90. RMSR was calculated as the root mean square radial distance of carpal centroids from the global centroid:

(2) $$RMSR=\sqrt{\frac{1}{n}\sum_{k=1}^{n}{∥c_{k}-\mu ∥}^{2}}.$$

MPD was calculated as the mean pairwise Euclidean distance between all carpal centroids:

(3) $$MPD=\frac{1}{n(n-1)}\sum_{\begin{array}{c} i,j=1\\ i\neq j \end{array}}^{n}∥c_{i}-c_{j}∥.$$

R50 and R90 were defined as the 50th and 90th percentiles of the radial distance distribution from the global centroid, respectively:

(4) $$R50=Q_{0.5}\left(\left\{∥c_{k}-\mu ∥:k=1,\ldots ,n\right\}\right),\;R90=Q_{0.9}\left(\left\{∥c_{k}-\mu ∥:k=1,\ldots ,n\right\}\right).$$

These metrics were investigated as potential quantitative imaging measures because they objectively summarize the global carpal spatial architecture from standard wrist radiographs. Higher values indicate greater spatial separation of the carpal bones, whereas lower values indicate a more compact carpal arrangement.

To evaluate longitudinal changes, annualized changes in each compactness metric were calculated as FU values minus BL values, divided by the follow-up duration in years. These annualized changes were subsequently used in correlation, subgroup, and multivariable regression analyses.

### 2.5. Statistical Analysis

Continuous variables are presented as mean ± standard deviation (SD). Annualized changes in AI-derived compactness metrics were calculated as the difference between FU and BL values divided by the follow-up duration in years.

Correlations between AI-derived compactness metrics and wrist radiographic scores were evaluated using Spearman’s rank correlation analysis at BL, FU, and for annualized changes. Spearman’s rank correlation analysis was also used to assess the relationships between BL ultrasound inflammatory metrics and BL compactness metrics, as well as between BL ultrasound inflammatory metrics and subsequent annualized changes in compactness metrics. Correlation coefficients (*r*) and corresponding *p* values were calculated.

Multivariable linear regression analyses were performed to identify independent associations between BL ultrasound inflammatory metrics and annualized changes in compactness metrics. BL ultrasound inflammatory metrics, BL compactness metrics, BL wrist radiographic scores, age, and sex were included as explanatory variables in the regression models. Sensitivity analyses were performed for all 16 ultrasound–compactness outcome combinations by additionally adjusting the primary multivariable models for either BL DAS28-ESR or BL DAS28-CRP in separate models. Within each set of 16 analyses, raw *p* values were adjusted using the Benjamini–Hochberg false discovery rate procedure.

Exploratory subgroup analyses were performed by classifying patients according to the presence or absence of BL GS and PD activity (score = 0 vs. score > 0). Annualized changes in compactness metrics were compared between groups using the Mann–Whitney U test.

All statistical analyses were performed using GraphPad Prism version 10 (GraphPad Software, San Diego, CA, USA). A two-sided *p* value < 0.05 was considered statistically significant.

## 3. Results

### 3.1. Relationship Between AI-Derived Compactness Metrics and Conventional Wrist Radiographic Scores

The correlations between AI-derived compactness metrics and conventional wrist radiographic scores were evaluated at BL, FU, and for annualized changes. Overall, no significant correlations were observed between compactness metrics and wrist radiographic scores (Table 2). All correlations remained non-significant after Benjamini–Hochberg FDR correction (all q≥0.420).

At BL, RMSR (*r* = 0.115, *p* = 0.151), MPD (*r* = 0.116, *p* = 0.145), R50 (*r* = 0.105, *p* = 0.190), and R90 (*r* = 0.091, *p* = 0.255) showed no significant correlations with wrist radiographic scores. Similar findings were observed at FU, with no significant correlations between any compactness metric and FU wrist radiographic scores.

Furthermore, annualized changes in compactness metrics were not significantly correlated with annualized changes in wrist radiographic scores (all *p* > 0.05).

### 3.2. Baseline Ultrasound Inflammation and Baseline Compactness Metrics

The correlations between BL ultrasound inflammatory metrics and BL AI-derived compactness metrics were evaluated. No significant correlations were observed between BL ultrasound inflammatory metrics and BL compactness metrics (Table 3). All associations remained non-significant after Benjamini–Hochberg FDR correction (all q≥0.381).

Neither GS, PD, GS+PD, nor VS% demonstrated significant correlations with RMSR, MPD, R50, or R90 at BL (all raw p>0.05). Detailed correlation coefficients, 95% confidence intervals, raw *p* values, and FDR-adjusted *q* values are presented in Table 3.

### 3.3. Baseline Ultrasound Inflammation and Longitudinal Changes in AI-Derived Compactness Metrics

BL ultrasound inflammatory metrics demonstrated significant positive correlations with annualized changes in all four AI-derived compactness metrics (Table 4). All associations remained statistically significant after Benjamini–Hochberg FDR correction, with FDR-adjusted *q* values ranging from 0.0045 to 0.0230.

For GS, significant positive correlations were observed with ΔRMSR/year (*r* = 0.245, *p* = 0.002), ΔMPD/year (*r* = 0.246, *p* = 0.002), ΔR50/year (*r* = 0.192, *p* = 0.015), and ΔR90/year (*r* = 0.251, *p* = 0.001). Similar significant positive correlations were observed for PD, GS+PD, and VS% across all four annualized compactness metric changes.

**Figure 3:**
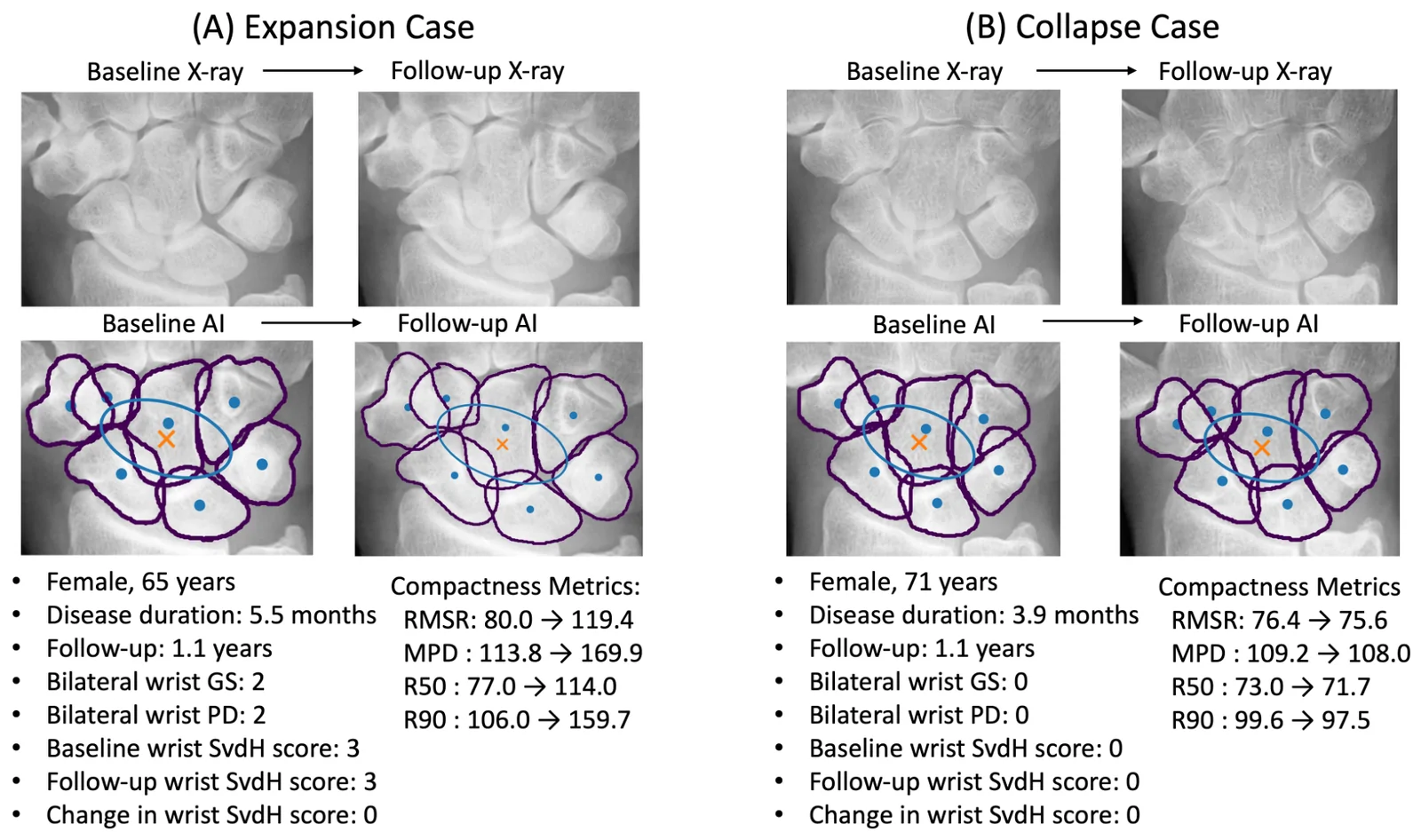
Representative examples of longitudinal changes captured by AI-derived compactness metrics. (**A**) Spatial expansion case. BL and FU radiographs, AI segmentation results, centroid distributions, and compactness metrics are shown. Despite an unchanged wrist radiographic score, increases were observed in RMSR, MPD, R50, and R90. (**B**) Collapse case. BL and FU radiographs, AI segmentation results, centroid distributions, and compactness metrics are shown. Despite a stable wrist radiographic score, decreases were observed in RMSR, MPD, R50, and R90. In the AI panels, the purple contours delineate the AI-segmented carpal bones, the blue dots represent the centroids of individual carpal bones, the orange cross represents the global centroid, and the light-blue ellipse illustrates the overall spatial distribution of the carpal-bone centroids. GS, gray-scale synovitis; PD, power Doppler synovitis; SvdH, Sharp/van der Heijde score; RMSR, root mean square radius; MPD, mean pairwise distance; R50, median radius; R90, 90th-percentile radius.

Representative examples of the longitudinal changes captured by the AI-derived compactness metrics are shown in Figure 3. Additional geometric visualizations of these representative cases, including convex hull analysis, ellipse-based analysis, and pairwise centroid distance heatmaps, are provided in Supplementary Figures S1–S3.

Among the four AI-derived compactness metrics, ΔR90/year showed relatively consistent correlations with BL ultrasound inflammatory metrics, including GS (*r* = 0.251, *p* = 0.001), PD (*r* = 0.242, *p* = 0.002), GS+PD (*r* = 0.245, *p* = 0.002), and VS% (*r* = 0.247, *p* = 0.002).

**Figure 4:**
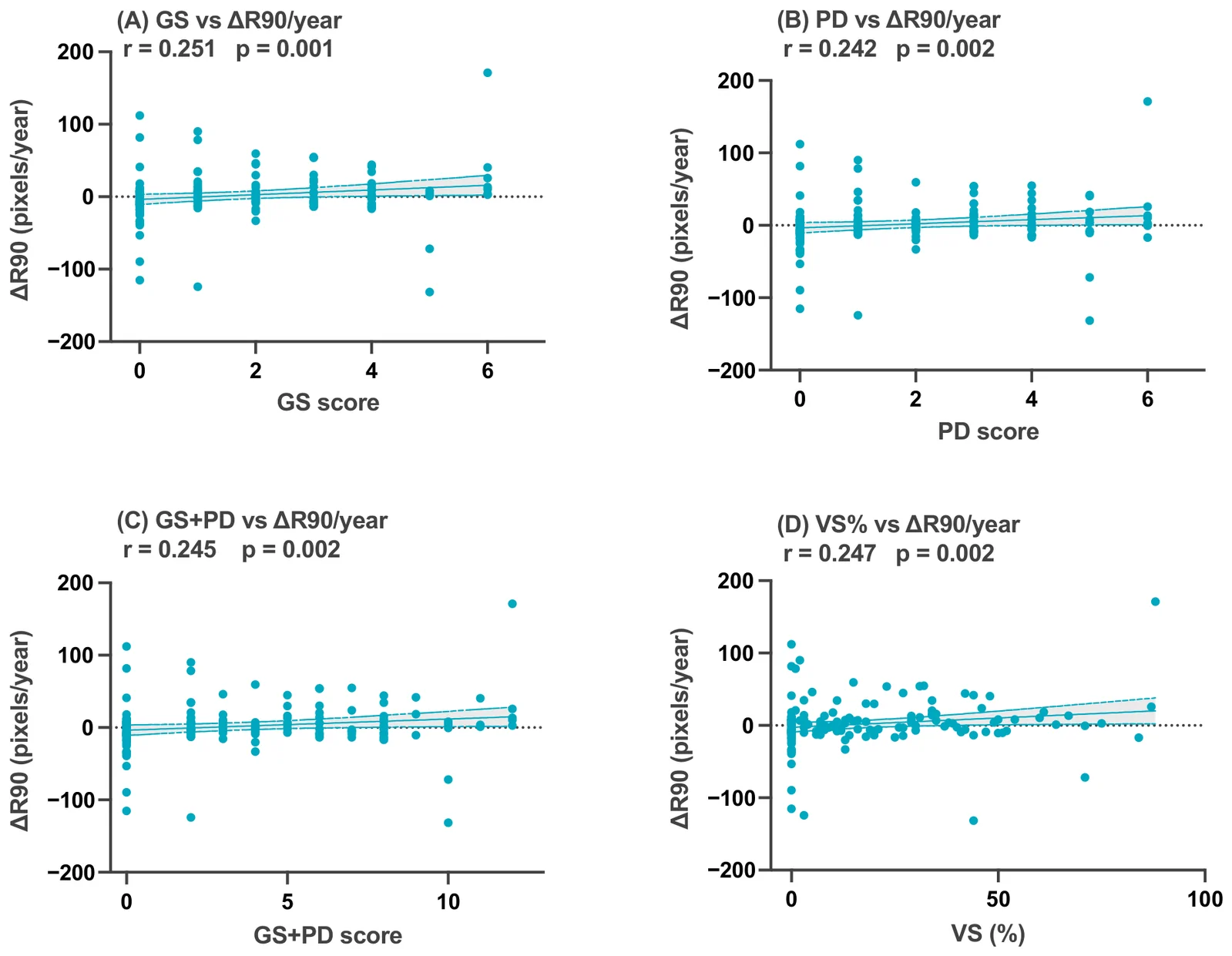
Correlations between BL ultrasound inflammatory metrics and annualized changes in R90. Scatter plots showing the correlations of BL GS (**A**), PD (**B**), GS+PD (**C**), and VS% (**D**) with annualized changes in the AI-derived compactness metric R90. Solid lines indicate linear regression fits and dashed lines indicate the 95% confidence intervals. Spearman correlation coefficients (*r*) and corresponding *p* values are shown in each panel. Annualized changes in the metrics are reported in pixels per year (where 1 pixel corresponds to a physical dimension of 0.15 mm).

Representative scatter plots illustrating the correlations between BL ultrasound inflammatory metrics and annualized changes in R90 are shown in Figure 4. Complete scatter plots for all AI-derived compactness metrics are provided in Supplementary Figures S4–S7.

### 3.4. Multivariable Regression Analysis

Multivariable regression analyses were performed to determine whether BL ultrasound inflammatory metrics were independently associated with annualized changes in AI-derived compactness metrics after adjustment for BL compactness metrics, BL wrist radiographic scores, age, and sex.

BL ultrasound inflammatory metrics remained significantly associated with annualized changes in several compactness metrics in the primary multivariable models after adjustment for these covariates (Table 5). The adjusted $R^{2}$ values of the multivariable models ranged from 0.100 to 0.280 (Table 6), indicating that the models explained a modest proportion of the variability in annualized compactness changes. Considering the imaging resolution (0.15 mm/pixel), these regression coefficients correspond to approximate annual changes of 0.1–0.6 mm, indicating subtle but measurable alterations in global carpal spatial architecture. For GS, significant independent associations were observed with annualized changes in RMSR (β = 2.447, raw *p* = 0.011), MPD (β = 3.363, raw *p* = 0.009), R50 (β = 1.917, raw *p* = 0.020), and R90 (β = 3.733, raw *p* = 0.006). All four GS associations remained statistically significant after Benjamini–Hochberg FDR correction (*q* = 0.0400 for all). Overall, 13 of the 16 associations remained statistically significant after FDR correction. For PD, the associations with RMSR, MPD, and R90 remained significant, whereas the association with R50 was non-significant (*q* = 0.0757). All four GS+PD associations remained significant. For VS%, the associations with MPD and R90 remained significant, whereas those with RMSR (*q* = 0.0559) and R50 (*q* = 0.0757) were non-significant.

Among the four compactness metrics, annualized changes in R90 demonstrated the most consistent independent associations with BL ultrasound inflammatory metrics, including GS (β = 3.733, raw *p* = 0.006, *q* = 0.0400), PD (β = 2.986, raw *p* = 0.019, *q* = 0.0400), GS+PD (β = 1.720, raw *p* = 0.010, *q* = 0.0400), and VS% (β = 0.253, raw *p* = 0.024, *q* = 0.0432).

A representative multivariable regression model evaluating the association between BL GS+PD and annualized changes in R90 is shown in Table 7. After adjustment for BL R90, BL wrist radiographic scores, age, and sex, BL GS+PD remained significantly associated with annualized changes in R90 (β = 1.720, 95% CI 0.422–3.018, raw *p* = 0.010, FDR-adjusted *q* = 0.0400). In contrast, BL wrist radiographic scores were not significantly associated with annualized changes in R90 (β = 0.821, 95% CI −1.401–3.044, *p* = 0.467).

In sensitivity analyses additionally adjusted for BL DAS28-ESR or BL DAS28-CRP, most associations between BL ultrasound inflammatory metrics and annualized changes in compactness metrics were attenuated. In both sets of models, the associations of BL GS with annualized changes in R50 and R90 remained significant based on raw *p* values; however, none of the 16 associations remained statistically significant after Benjamini–Hochberg FDR correction. Complete results are presented in Supplementary Tables S1 and S2.

### 3.5. Exploratory Subgroup Analysis

Exploratory subgroup analyses were performed by classifying patients according to the presence or absence of BL GS and PD activity. Patients with detectable BL GS activity (GS > 0, *n* = 101) demonstrated significantly higher annualized changes in all compactness metrics than those without GS activity (GS = 0, *n* = 58). Similar findings were observed for PD-positive (*n* = 102) and PD-negative (*n* = 57) patients (Table 8). All eight subgroup comparisons remained statistically significant after Benjamini–Hochberg FDR correction, with adjusted *q* values ranging from 0.0016 to 0.0073.

For ΔR90/year, the median value was −6.37 in the GS-negative group and 3.09 in the GS-positive group (raw *p* = 0.0004, *q* = 0.0016). For ΔRMSR/year, the median value was −2.63 in the GS-negative group and 0.68 in the GS-positive group (raw *p* = 0.0016, *q* = 0.0023).

Similarly, the median ΔR90/year was −6.87 in the PD-negative group and 3.04 in the PD-positive group (raw *p* = 0.0004, *q* = 0.0016), whereas the median ΔRMSR/year was −2.66 and 0.68, respectively (raw *p* = 0.0016, *q* = 0.0023). Significant between-group differences were also observed for ΔMPD/year and ΔR50/year.

## 4. Discussion

### 4.1. Principal Findings

The principal contribution of this study is the evaluation of AI-derived centroid-based compactness metrics as potential quantitative imaging measures of global carpal configuration in patients with early RA. Unlike conventional radiographic scoring, which mainly reflects established local structural damage such as BE and JSN [23,24], these metrics objectively summarize the spatial distribution of multiple carpal bones on standard wrist radiographs.

The main findings were as follows. First, BL and FU compactness metrics were not significantly associated with conventional wrist radiographic scores. Second, longitudinal changes in compactness metrics were not significantly associated with changes in manual wrist scores. Third, BL ultrasound inflammation was not clearly associated with BL compactness values, but higher BL GS and PD activity was significantly associated with subsequent increases in compactness metrics. These findings suggest that AI-derived compactness metrics may provide additional structural information on inflammation-associated changes in global carpal spatial architecture that are not reflected by conventional radiographic scoring.

### 4.2. Complementary Role of AI-Derived Compactness Metrics

The absence of significant associations between AI-derived compactness metrics and conventional wrist radiographic scores suggests that these two approaches may reflect different aspects of wrist involvement in early RA. Wrist SvdH scoring is designed to quantify established structural damage, including BE and JSN, at predefined anatomical sites [7]. In contrast, centroid-based compactness metrics summarize the global spatial configuration of multiple carpal bones. Therefore, the two approaches should not be regarded as directly interchangeable.

This distinction is particularly relevant in early RA. At this disease stage, manual radiographic scores may remain low or show little progression during short-term FU, even when active inflammation is present. As a result, subtle changes in carpal alignment or intercarpal spatial relationships may occur without producing a measurable increase in conventional radiographic scores. The lack of association between annualized changes in compactness metrics and annualized changes in manual wrist scores supports this interpretation.

Thus, compactness metrics should not be regarded as a replacement for conventional radiographic scoring. Rather, they may serve as complementary, objective quantitative tools for describing a different dimension of wrist involvement: global carpal spatial architecture [25].

### 4.3. Potential Anatomical and Biological Interpretation

The longitudinal association between BL ultrasound inflammation and subsequent increases in compactness metrics suggests that active synovitis may influence the spatial organization of the carpus during the early phase of RA. Rather than representing established structural damage, increases in compactness metrics may reflect inflammation-associated spatial expansion or reconfiguration of carpal spatial architecture. These annual changes corresponded to only approximately 0.1–0.6 mm; however, such subtle changes are expected in early RA and may reflect inflammation-related alterations that are not readily detectable by conventional radiographic scoring. Although several associations remained statistically significant after multivariable adjustment, the modest adjusted $R^{2}$ values (0.100–0.280) indicate that a substantial proportion of the variability in longitudinal compactness changes remains unexplained. Therefore, baseline ultrasound inflammation should be regarded as one of multiple potential contributors rather than a strong determinant of subsequent carpal geometric change.

One possible explanation is that active synovitis and inflammation-related periarticular soft tissue changes may affect the spatial relationship among carpal bones before definite radiographic damage becomes apparent [26]. In the early phase of RA, synovial hypertrophy or joint effusion may be associated with subtle changes in carpal alignment or intercarpal spacing [26,27], resulting in mild separation or redistribution of carpal bone centroids. Because RMSR, MPD, R50, and R90 summarize the relative spatial distribution of multiple carpal bones, these metrics may be sensitive to such global architectural changes even when conventional radiographic scores remain unchanged.

The absence of a clear association between BL ultrasound inflammation and BL compactness values further supports this dynamic interpretation. BL compactness values may be influenced by individual anatomical variation, radiographic positioning, and pre-existing differences in carpal alignment. Importantly, the AI pipeline used in this study is deterministic, such that repeated analysis of the same radiograph always yields identical compactness metrics. Consequently, any remaining measurement variability is more likely to arise from radiographic acquisition conditions than from the AI algorithm itself. In contrast, longitudinal changes in compactness metrics may more directly reflect remodeling processes that develop over time. Thus, BL ultrasound inflammation may be more closely related to subsequent architectural change than to static carpal configuration at a single time point. The attenuation of several associations after additional adjustment for BL DAS28-ESR or BL DAS28-CRP suggests that part of the observed relationship may be explained by the overall baseline inflammatory burden.

### 4.4. Stage-Dependent Changes in Carpal Architecture

The present findings may also be considered in the context of stage-dependent changes in carpal architecture in RA. In early RA, active inflammation may be associated with subtle spatial expansion or reconfiguration of carpal spatial organization, as reflected by increases in centroid-based compactness metrics. In contrast, in established or more advanced RA, longstanding inflammation leads to progressive cartilage loss, erosive destruction, and carpal collapse, which manifest as radiographic JSN and reduced intercarpal distances, ultimately leading to a more compact carpal configuration [28].

This interpretation is consistent with our previous study, in which more severe radiographic JSN was associated with smaller centroid-based distance metrics in an RA cohort with more established structural damage [19]. However, this stage-dependent interpretation remains hypothetical, and the biological meaning of changes in compactness should be validated in future longitudinal studies that include patients across different RA stages. In addition, investigating the relationship between compactness metrics and long-term structural progression and clinical outcomes will be important to further validate this proposed hypothesis and better define its biological significance.

### 4.5. Limitations and Future Perspectives

Several limitations should be acknowledged. First, this was a retrospective single-center study involving Japanese patients with early RA. In addition, radiographs with severe structural destruction, extensive bony ankylosis, or advanced erosive changes unsuitable for reliable AI-based compactness analysis were excluded from this study. Therefore, the present findings are primarily applicable to patients with early RA and relatively preserved carpal anatomy, and their generalizability to other populations, healthcare settings, and patients with advanced-stage RA remains to be established. External validation in independent cohorts is therefore required. Second, ultrasound inflammation scores were analyzed as bilateral wrist totals, which limited side-specific evaluation of the relationship between ultrasound findings and radiographic compactness changes. Inter-observer agreement was not reassessed in this retrospective cohort because duplicate independent ultrasound evaluations were not available. Therefore, study-specific ICC or Cohen’s kappa statistics could not be calculated. Nevertheless, the same standardized acquisition protocol and observer-training framework demonstrated excellent inter-observer agreement in our previous studies [21,22]. Third, compactness metrics were derived from two-dimensional radiographs and therefore may not fully represent three-dimensional carpal morphology or alignment. Although centroid localization accuracy was not directly evaluated, the AP-DPM framework has previously demonstrated excellent segmentation performance (Dice coefficient ≈ 0.98). Therefore, substantial centroid localization errors are considered unlikely. Fourth, although these metrics may reflect inflammation-associated changes in carpal architecture, their biological interpretation remains uncertain. Further validation using complementary anatomical imaging, such as MRI or CT, is required. Finally, the clinical significance of increased compactness metrics remains to be clarified, particularly in relation to symptoms, functional outcomes, treatment response, and long-term radiographic progression. Although the sensitivity analyses additionally adjusted for BL DAS28-ESR and BL DAS28-CRP, residual confounding by other clinical factors, including disease duration, serological status, medication use, and treatment changes during follow-up, cannot be excluded. These factors should be evaluated in future prospective studies with larger cohorts and more comprehensive longitudinal clinical data.

Future studies with larger multicenter cohorts, longer follow-up periods, side-specific ultrasound assessment, and comprehensive longitudinal clinical and outcome data are needed to validate these findings. Because the AI pipeline used in this study is deterministic, repeated analysis of the same radiograph always yields identical compactness metrics. In addition, future studies should evaluate acquisition-related repeatability and positioning sensitivity using repeated radiographic examinations obtained under standardized imaging conditions. Accordingly, the present findings should be regarded as hypothesis-generating until further external and methodological validation is completed.

## 5. Conclusions

AI-derived centroid-based compactness metrics may provide complementary quantitative information on global carpal spatial architecture in patients with early RA. These metrics were not significantly associated with conventional wrist radiographic scores, whereas their annualized changes were associated with BL ultrasound inflammation, suggesting that they provide complementary information beyond conventional radiographic assessment. AI-derived compactness metrics may therefore offer an objective quantitative approach for evaluating inflammation-associated changes in carpal architecture on standard wrist radiographs.

## Figures and Tables

**Table 1:** Demographic, clinical, ultrasound, radiographic, and AI-derived compactness metric characteristics of the study cohort.

Characteristic	Value
**Patients**	
Number of patients	159
Age, years	60.1±14.8
Female, n (%)	114 (71.7%)
Follow-up duration, years	1.3±0.5
**BL ultrasound parameters**	
BL GS	1.7±1.8
BL PD	1.9±1.9
BL GS+PD	3.5±3.6
BL VS (%)	17.0±21.4
**Manual wrist score**	
BL	1.2±2.1
FU	1.8±2.7
**AI-derived compactness metrics**	
RMSR (BL)	178.9±22.2
RMSR (FU)	180.3±26.5
MPD (BL)	251.6±28.9
MPD (FU)	253.5±35.8
R50 (BL)	168.2±18.3
R50 (FU)	170.1±23.9
R90 (BL)	238.6±31.5
R90 (FU)	240.5±36.4

All AI-derived compactness metrics are presented in pixels. Because all radiographs were acquired using the same imaging protocol (pixel size = 0.15 mm), one pixel corresponds to approximately 0.15 mm. Accordingly, annual changes of 2–4 pixels correspond to physical changes of approximately 0.3–0.6 mm.

**Table 2:** Correlations between AI-derived compactness metrics and wrist radiographic scores.

Comparison	Metric	*r*	95% CI	Raw *p*	FDR-Adjusted *q*
BL	RMSR	0.115	−0.046 to 0.270	0.151	0.420
	MPD	0.116	−0.045 to 0.271	0.145	0.420
	R50	0.105	−0.057 to 0.260	0.190	0.420
	R90	0.091	−0.070 to 0.247	0.255	0.420
FU	RMSR	0.089	−0.073 to 0.245	0.266	0.420
	MPD	0.086	−0.075 to 0.243	0.280	0.420
	R50	0.063	−0.098 to 0.221	0.429	0.468
	R90	0.092	−0.069 to 0.249	0.248	0.420
Annualized change	RMSR	0.063	−0.098 to 0.221	0.429	0.468
	MPD	0.072	−0.089 to 0.229	0.368	0.468
	R50	0.011	−0.149 to 0.171	0.889	0.889
	R90	0.089	−0.073 to 0.245	0.267	0.420

Raw *p* values were adjusted using the Benjamini–Hochberg false discovery rate procedure within this family of 12 correlation analyses. An FDR-adjusted *q* value <0.05 was considered statistically significant.

**Table 3:** Correlations between BL ultrasound inflammatory metrics and BL AI-derived compactness metrics.

Ultrasound Parameter	AI Metric	*r*	95% CI	Raw *p*	FDR-Adjusted *q*
GS	RMSR	0.099	−0.062 to 0.255	0.213	0.381
	MPD	0.097	−0.065 to 0.253	0.226	0.381
	R50	0.136	−0.025 to 0.290	0.088	0.381
	R90	0.109	−0.052 to 0.265	0.170	0.381
PD	RMSR	0.079	−0.082 to 0.236	0.321	0.381
	MPD	0.077	−0.084 to 0.235	0.334	0.381
	R50	0.112	−0.050 to 0.267	0.162	0.381
	R90	0.091	−0.070 to 0.247	0.255	0.381
GS+PD	RMSR	0.088	−0.074 to 0.244	0.273	0.381
	MPD	0.085	−0.076 to 0.242	0.286	0.381
	R50	0.122	−0.039 to 0.276	0.127	0.381
	R90	0.100	−0.062 to 0.256	0.212	0.381
VS%	RMSR	0.069	−0.092 to 0.227	0.386	0.410
	MPD	0.066	−0.095 to 0.224	0.410	0.410
	R50	0.094	−0.067 to 0.250	0.239	0.381
	R90	0.086	−0.075 to 0.243	0.280	0.381

Raw *p* values were adjusted using the Benjamini–Hochberg false discovery rate procedure within this family of 16 correlation analyses. An FDR-adjusted *q* value <0.05 was considered statistically significant.

**Table 4:** Correlations between BL ultrasound inflammatory parameters and annualized changes in AI-derived compactness metrics.

Ultrasound Parameter	Metric Change	*r*	95% CI	Raw *p*	FDR-Adjusted *q*
GS	ΔRMSR/year	0.245	0.088 to 0.390	0.002	0.0045
	ΔMPD/year	0.246	0.089 to 0.391	0.002	0.0045
	ΔR50/year	0.192	0.033 to 0.342	0.015	0.0188
	ΔR90/year	0.251	0.094 to 0.395	0.001	0.0045
PD	ΔRMSR/year	0.234	0.076 to 0.380	0.003	0.0045
	ΔMPD/year	0.232	0.075 to 0.378	0.003	0.0045
	ΔR50/year	0.180	0.021 to 0.331	0.023	0.0230
	ΔR90/year	0.242	0.085 to 0.387	0.002	0.0045
GS+PD	ΔRMSR/year	0.238	0.081 to 0.383	0.003	0.0045
	ΔMPD/year	0.237	0.080 to 0.383	0.003	0.0045
	ΔR50/year	0.184	0.024 to 0.334	0.020	0.0227
	ΔR90/year	0.245	0.088 to 0.390	0.002	0.0045
VS%	ΔRMSR/year	0.234	0.077 to 0.380	0.003	0.0045
	ΔMPD/year	0.231	0.074 to 0.378	0.003	0.0045
	ΔR50/year	0.183	0.023 to 0.333	0.021	0.0227
	ΔR90/year	0.247	0.090 to 0.391	0.002	0.0045

Raw *p* values were adjusted using the Benjamini–Hochberg false discovery rate procedure within this family of 16 correlation analyses. An FDR-adjusted *q* value <0.05 was considered statistically significant.

**Table 5:** Summary of multivariable regression models evaluating the independent associations between BL ultrasound inflammatory metrics and annualized changes in AI-derived compactness metrics after adjustment for BL compactness metrics, BL wrist radiographic scores, age, and sex.

BL Ultrasound Metric	ΔRMSR/Year	ΔMPD/Year	ΔR50/Year	ΔR90/Year
	β (95% CI), Raw p; FDR q	β (95% CI), Raw p; FDR q	β (95% CI), Raw p; FDR q	β (95% CI), Raw p; FDR q
GS	2.447 (0.576–4.318), 0.011; *q* = 0.0400	3.363 (0.861–5.865), 0.009; *q* = 0.0400	1.917 (0.307–3.528), 0.020; *q* = 0.0400	3.733 (1.101–6.365), 0.006; *q* = 0.0400
PD	1.880 (0.109–3.651), 0.038; *q* = 0.0469	2.604 (0.234–4.973), 0.032; *q* = 0.0469	1.378 (−0.144–2.900), 0.076; *q* = 0.0757	2.986 (0.497–5.475), 0.019; *q* = 0.0400
GS+PD	1.106 (0.183–2.030), 0.019; *q* = 0.0400	1.526 (0.291–2.761), 0.016; *q* = 0.0400	0.841 (0.047–1.635), 0.038; *q* = 0.0469	1.720 (0.422–3.018), 0.010; *q* = 0.0400
VS%	0.157 (0.001–0.313), 0.049; *q* = 0.0559	0.221 (0.012–0.430), 0.038; *q* = 0.0469	0.122 (−0.012–0.256), 0.074; *q* = 0.0757	0.253 (0.033–0.472), 0.024; *q* = 0.0432

Raw *p* values for the associations of BL ultrasound inflammatory metrics with the four annualized compactness outcomes were adjusted using the Benjamini–Hochberg false discovery rate procedure as one family of 16 tests. FDR-adjusted *q* values were calculated using the unrounded raw *p* values, and q<0.05 was considered statistically significant. Adjusted $R^{2}$ values for the corresponding multivariable models are presented separately in Table 6.

**Table 6:** Adjusted $R^{2}$ values for the multivariable regression models evaluating the associations between baseline ultrasound inflammatory metrics and annualized changes in AI-derived compactness metrics.

BL Ultrasound Metric	ΔRMSR/Year	ΔMPD/Year	ΔR50/Year	ΔR90/Year
GS	0.250	0.208	0.113	0.280
PD	0.239	0.196	0.100	0.270
GS+PD	0.245	0.202	0.107	0.276
VS%	0.237	0.194	0.100	0.268

Adjusted $R^{2}$ indicates the proportion of variance explained by each multivariable regression model after accounting for the number of predictors. Each model included one baseline ultrasound inflammatory metric and was adjusted for the corresponding baseline AI-derived compactness metric, baseline wrist radiographic score, age, and sex.

**Table 7:** Representative multivariable regression model evaluating the association between BL GS+PD and annualized changes in R90.

Variable	β	95% CI	*p*
GS+PD	1.720	0.422 to 3.018	0.010
BL R90	−0.560	−0.713 to −0.407	<0.0001
BL wrist radiographic score	0.821	−1.401 to 3.044	0.467
Sex	−25.16	−35.81 to −14.51	<0.0001
Age	−0.086	−0.402 to 0.230	0.591

**Table 8:** Exploratory subgroup analyses of annualized changes in AI-derived compactness metrics according to BL ultrasound inflammatory activity. Values are presented as medians.

Metric	GS Activity				PD Activity			
	GS = 0 (n = 58)	GS > 0 (n = 101)	Raw p	FDR-Adjusted q	PD = 0 (n = 57)	PD > 0 (n = 102)	Raw p	FDR-Adjusted q
ΔRMSR/year	−2.631	0.682	0.0016	0.0023	−2.657	0.677	0.0016	0.0023
ΔMPD/year	−3.085	0.727	0.0016	0.0023	−3.182	0.562	0.0017	0.0023
ΔR50/year	−2.224	1.379	0.0073	0.0073	−2.263	1.399	0.0049	0.0056
ΔR90/year	−6.368	3.086	0.0004	0.0016	−6.872	3.040	0.0004	0.0016

Raw *p* values were adjusted using the Benjamini–Hochberg false discovery rate procedure within this exploratory family of eight subgroup comparisons. All comparisons remained statistically significant after FDR correction (q<0.05).

## Data Availability

The imaging datasets generated and analyzed during the current study are not publicly available because they contain potentially identifiable patient information and are subject to institutional ethics and privacy regulations. De-identified data may be made available from the corresponding author upon reasonable request, subject to approval by the institutional review board and applicable data-sharing regulations.

## Supplementary Materials

The following supporting information can be downloaded at: https://www.mdpi.com/article/10.3390/jimaging12080363/s1, Figure S1: Convex hull visualization of representative spatial expansion and collapse cases. (A) Representative spatial expansion case. (B) Representative collapse case. Convex hulls derived from carpal bone centroids are shown at BL, FU, and as overlay visualizations. Figure S2: Ellipse-based visualization of representative spatial expansion and collapse cases. (A) Representative spatial expansion case. (B) Representative collapse case. Ellipses fitted to the carpal bone centroid distribution are shown at BL, FU, and as overlay visualizations. Figure S3: Pairwise centroid distance heatmaps of representative spatial expansion and collapse cases. The difference matrix represents FU minus BL values. Red indicates increased intercarpal centroid distance, whereas blue indicates decreased distance. Figure S4: Correlations between BL GS scores and annualized changes in AI-derived compactness metrics. Scatter plots showing the relationships between BL GS scores and annualized changes in RMSR (A), MPD (B), R50 (C), and R90 (D). Spearman correlation coefficients (*r*) and corresponding *p* values are shown in each panel. Annualized changes in the metrics are reported in pixels per year (where 1 pixel corresponds to a physical dimension of 0.15 mm). Figure S5: Correlations between BL PD scores and annualized changes in AI-derived compactness metrics. Scatter plots showing the relationships between BL PD scores and annualized changes in RMSR (A), MPD (B), R50 (C), and R90 (D). Spearman correlation coefficients (*r*) and corresponding *p* values are shown in each panel. Annualized changes in the metrics are reported in pixels per year (where 1 pixel corresponds to a physical dimension of 0.15 mm). Figure S6: Correlations between BL GS+PD scores and annualized changes in AI-derived compactness metrics. Scatter plots showing the relationships between BL GS+PD scores and annualized changes in RMSR (A), MPD (B), R50 (C), and R90 (D). Spearman correlation coefficients (*r*) and corresponding *p* values are shown in each panel. Annualized changes in the metrics are reported in pixels per year (where 1 pixel corresponds to a physical dimension of 0.15 mm). Figure S7: Correlations between BL VS% and annualized changes in AI-derived compactness metrics. Scatter plots showing the relationships between BL VS% and annualized changes in RMSR (A), MPD (B), R50 (C), and R90 (D). Spearman correlation coefficients (*r*) and corresponding *p* values are shown in each panel. Annualized changes in the metrics are reported in pixels per year (where 1 pixel corresponds to a physical dimension of 0.15 mm). Table S1: Sensitivity analyses of the associations between BL ultrasound inflammatory metrics and annualized changes in AI-derived compactness metrics after additional adjustment for BL DAS28-ESR. Table S2: Sensitivity analyses of the associations between BL ultrasound inflammatory metrics and annualized changes in AI-derived compactness metrics after additional adjustment for BL DAS28-CRP.
