# Supplementary material for: AI-Derived Carpal Compactness Metrics as Quantitative Imaging Measures of Global Carpal Architecture in Early Rheumatoid Arthritis
Source: J Imaging. 2026 Aug 8;12(8):363. doi: 10.3390/jimaging12080363 (PMC13514302; doi:10.3390/jimaging12080363)
Supplement: Supplementary file 1 [file jimaging-12-00363-s001.zip › fig/Supp_Figure_S4.pdf]

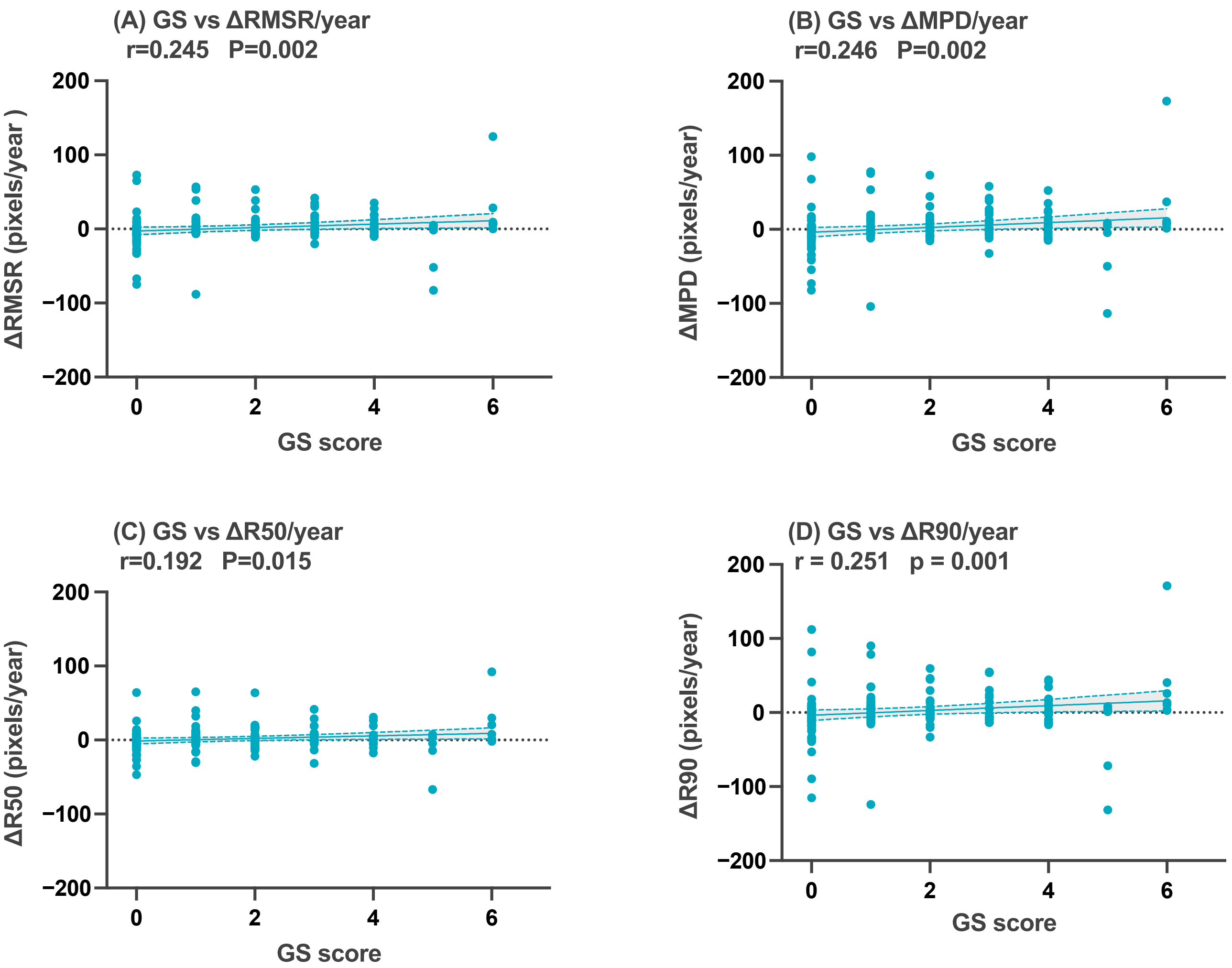

(A) GS vs ΔRMSR/year
r=0.245 P=0.002
ΔRMSR (pixels/year)
200
100
0
−100
−200
0
2
4
6
GS score
(B) GS vs ΔMPD/year
r=0.246 P=0.002
ΔMPD (pixels/year)
200
100
0
−100
−200
0
2
4
6
GS score
(C) GS vs ΔR50/year
r=0.192 P=0.015
ΔR50 (pixels/year)
200
100
0
−100
−200
0
2
4
6
GS score
(D) GS vs ΔR90/year
r = 0.251 p = 0.001
ΔR90 (pixels/year)
200
100
0
−100
−200
0
2
4
6
GS score
