# Supplementary material for: AI-Derived Carpal Compactness Metrics as Quantitative Imaging Measures of Global Carpal Architecture in Early Rheumatoid Arthritis
Source: J Imaging. 2026 Aug 8;12(8):363. doi: 10.3390/jimaging12080363 (PMC13514302; doi:10.3390/jimaging12080363)
Supplement: Supplementary file 1 [file jimaging-12-00363-s001.zip › fig/Supp_Figure_S5.pdf]

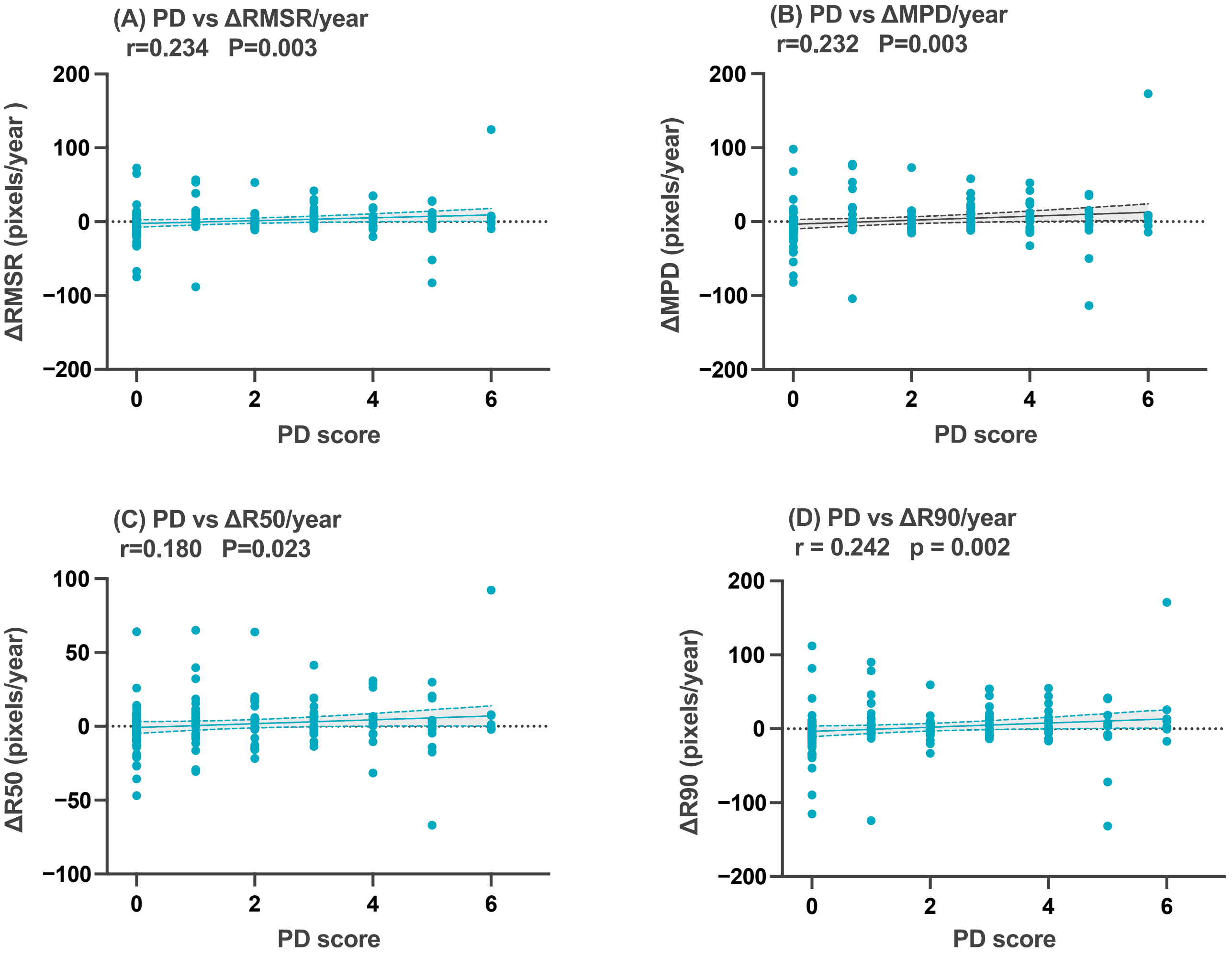

(A) PD vs ΔRMSR/year
r=0.234 P=0.003
ΔRMSR (pixels/year)
200
100
0
−100
−200
0
2
4
6
PD score
(B) PD vs ΔMPD/year
r=0.232 P=0.003
ΔMPD (pixels/year)
200
100
0
−100
−200
0
2
4
6
PD score
(C) PD vs ΔR50/year
r=0.180 P=0.023
ΔR50 (pixels/year)
100
50
0
−50
−100
0
2
4
6
PD score
(D) PD vs ΔR90/year
r = 0.242 p = 0.002
ΔR90 (pixels/year)
200
100
0
−100
−200
0
2
4
6
PD score
