# Supplementary material for: AI-Derived Carpal Compactness Metrics as Quantitative Imaging Measures of Global Carpal Architecture in Early Rheumatoid Arthritis
Source: J Imaging. 2026 Aug 8;12(8):363. doi: 10.3390/jimaging12080363 (PMC13514302; doi:10.3390/jimaging12080363)
Supplement: Supplementary file 1 [file jimaging-12-00363-s001.zip › fig/Supp_Figure_S6.pdf]

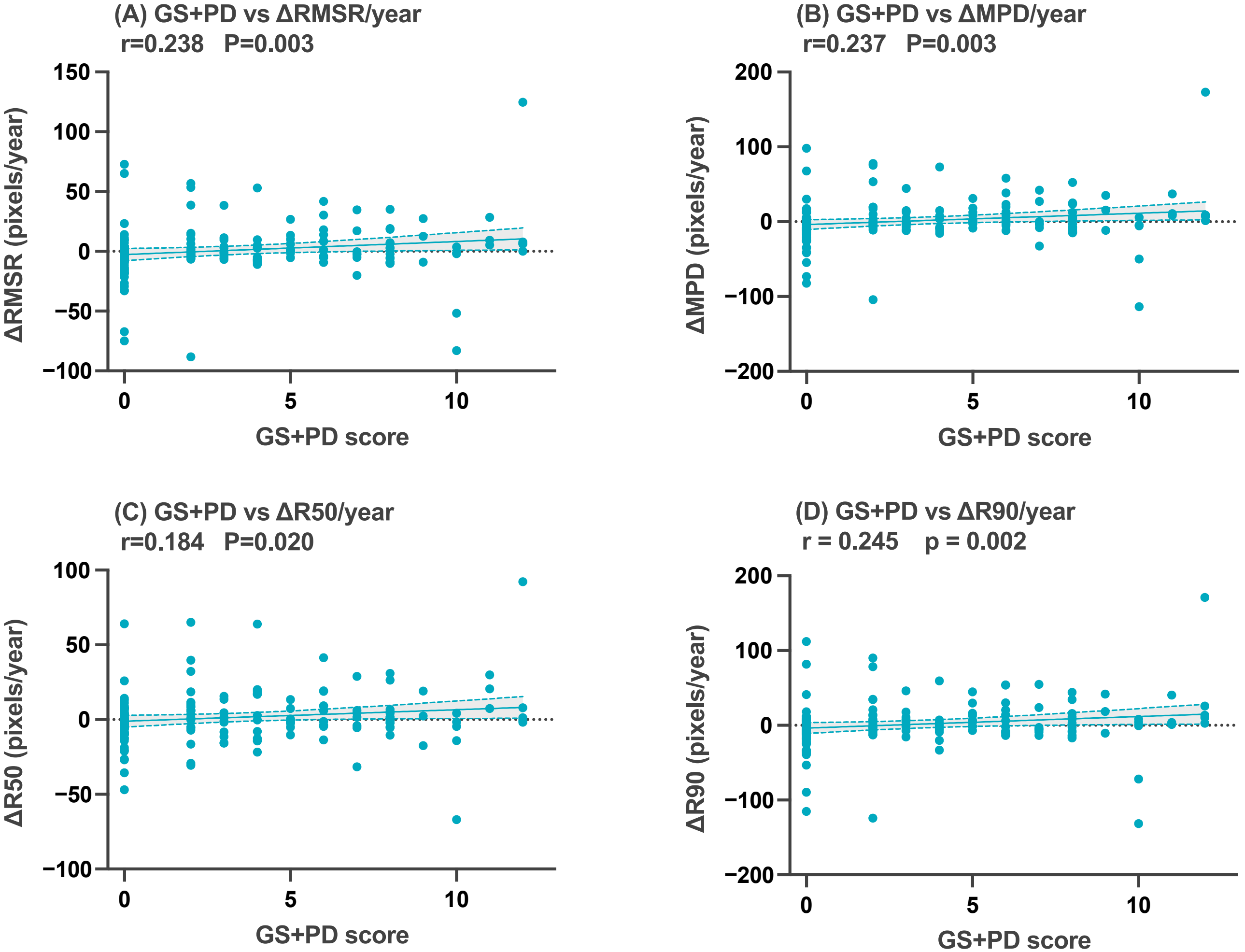
(A) GS+PD vs ΔRMSR/year
r=0.238 P=0.003
ΔRMSR (pixels/year)
150
100
50
0
−50
−100
0
5
10
GS+PD score
(B) GS+PD vs ΔMPD/year
r=0.237 P=0.003
ΔMPD (pixels/year)
200
100
0
−100
−200
0
5
10
GS+PD score
(C) GS+PD vs ΔR50/year
r=0.184 P=0.020
ΔR50 (pixels/year)
100
50
0
−50
−100
0
5
10
GS+PD score
(D) GS+PD vs ΔR90/year
r = 0.245 p = 0.002
ΔR90 (pixels/year)
200
100
0
−100
−200
0
5
10
GS+PD score
