# Supplementary material for: AI-Derived Carpal Compactness Metrics as Quantitative Imaging Measures of Global Carpal Architecture in Early Rheumatoid Arthritis
Source: J Imaging. 2026 Aug 8;12(8):363. doi: 10.3390/jimaging12080363 (PMC13514302; doi:10.3390/jimaging12080363)
Supplement: Supplementary file 1 [file jimaging-12-00363-s001.zip › fig/Supp_Figure_S7.pdf]

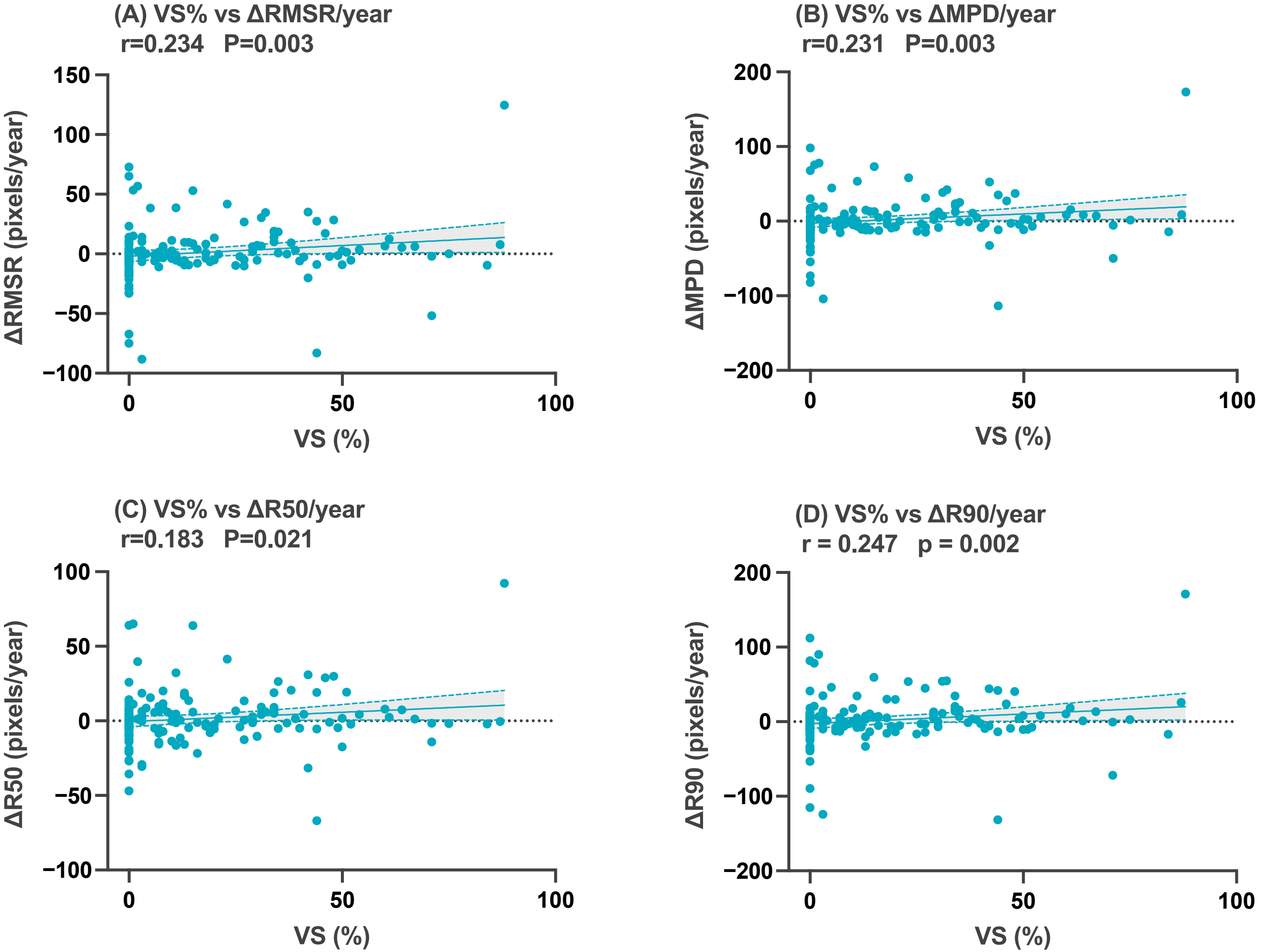

(A) VS% vs ΔRMSR/year
r=0.234 P=0.003
ΔRMSR (pixels/year)
150
100
50
0
−50
−100
0
50
100
VS (%)
(B) VS% vs ΔMPD/year
r=0.231 P=0.003
ΔMPD (pixels/year)
200
100
0
−100
−200
0
50
100
VS (%)
(C) VS% vs ΔR50/year
r=0.183 P=0.021
ΔR50 (pixels/year)
100
50
0
−50
−100
0
50
100
VS (%)
(D) VS% vs ΔR90/year
r = 0.247 p = 0.002
ΔR90 (pixels/year)
200
100
0
−100
−200
0
50
100
VS (%)
