# Supplementary material for: AI-Derived Carpal Compactness Metrics as Quantitative Imaging Measures of Global Carpal Architecture in Early Rheumatoid Arthritis
Source: J Imaging. 2026 Aug 8;12(8):363. doi: 10.3390/jimaging12080363 (PMC13514302; doi:10.3390/jimaging12080363)
Supplement: Supplementary file 1 [file jimaging-12-00363-s001.zip › Supplementary Materials.pdf]

Supplementary Materials: AI-Derived Carpal Compactness Metrics as Quantitative Imaging Measures of Global Carpal Architecture in Early Rheumatoid Arthritis

Jiajing Zhou [1], Haolin Wang [2,*], Ikuma Nakagawa [3], Shun Tanimura [3], Yuhei Shibata [3], Ken Nagahata [3] and Tamotsu Kamishima [1]

Supplementary Materials

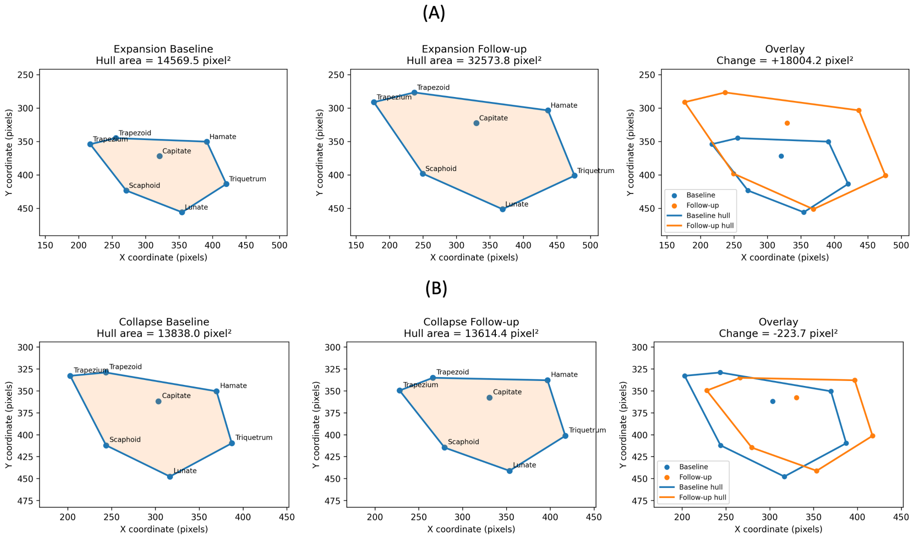

Figure S1. Convex hull visualization of representative spatial expansion and collapse cases. (A) Representative spatial expansion case. (B) Representative collapse case. Convex hulls derived from carpal bone centroids are shown at BL, FU, and as overlay visualizations.

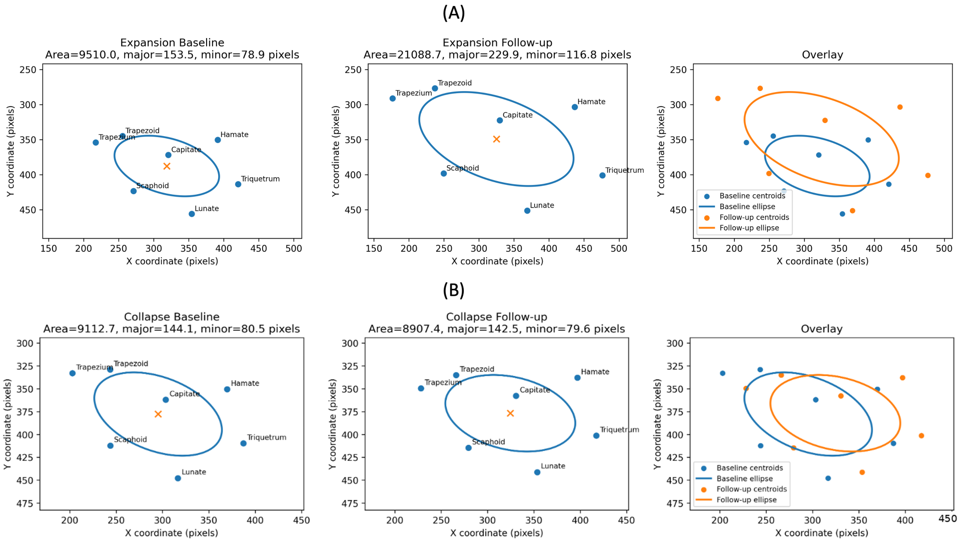

Figure S2. Ellipse-based visualization of representative spatial expansion and collapse cases. (A) Representative spatial expansion case. (B) Representative collapse case. Ellipses fitted to the carpal bone centroid distribution are shown at BL, FU, and as overlay visualizations.

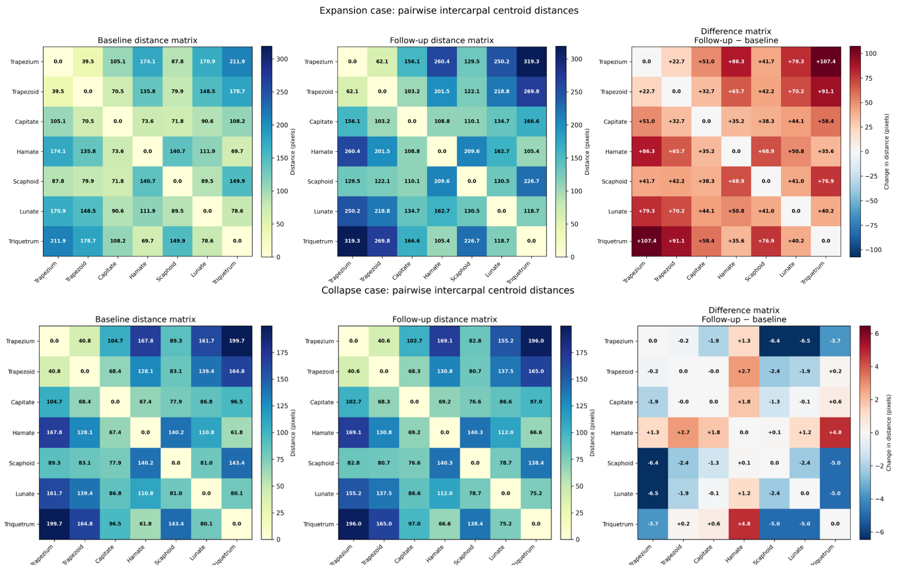

Figure S3. Pairwise centroid distance heatmaps of representative spatial expansion and collapse cases. The difference matrix represents FU minus BL values. Red indicates increased intercarpal centroid distance, whereas blue indicates decreased distance.

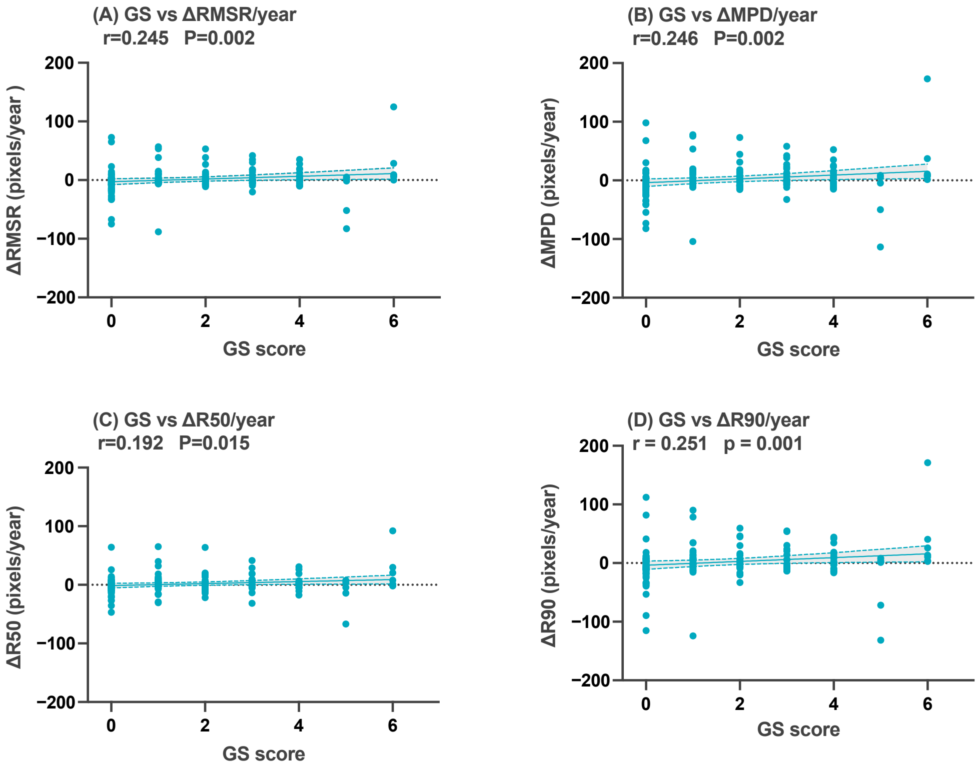

Figure S4. Correlations between BL GS scores and annualized changes in AI-derived compactness metrics. Scatter plots showing the relationships between BL GS scores and annualized changes in RMSR (A), MPD (B), R50 (C), and R90 (D). Spearman correlation coefficients (*r*) and corresponding *p* values are shown in each panel. Annualized changes in the metrics are reported in pixels per year (where 1 pixel corresponds to a physical dimension of 0.15 mm).

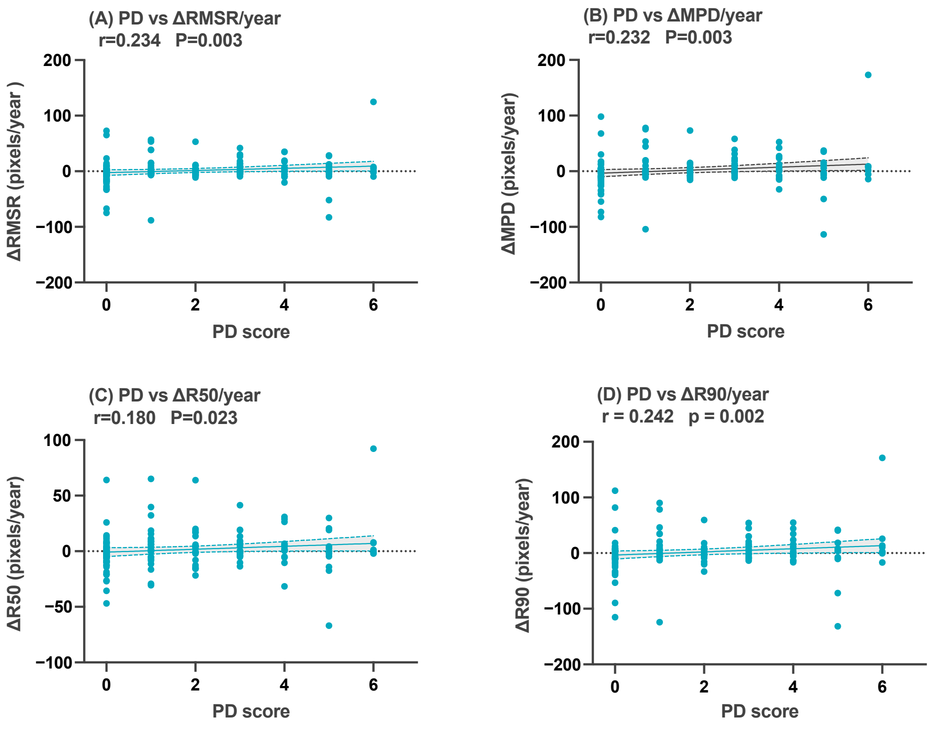

Figure S5. Correlations between BL PD scores and annualized changes in AI-derived compactness metrics. Scatter plots showing the relationships between BL PD scores and annualized changes in RMSR (A), MPD (B), R50 (C), and R90 (D). Spearman correlation coefficients (*r*) and corresponding *p* values are shown in each panel. Annualized changes in the metrics are reported in pixels per year (where 1 pixel corresponds to a physical dimension of 0.15 mm).

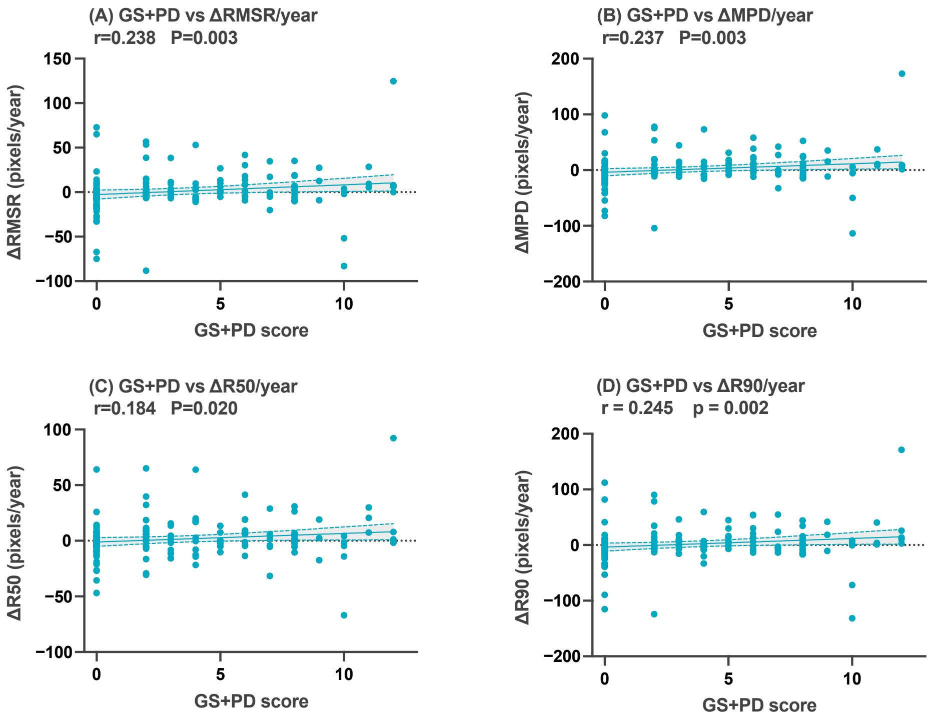

Figure S6. Correlations between BL GS+PD scores and annualized changes in AI-derived compactness metrics. Scatter plots showing the relationships between BL GS+PD scores and annualized changes in RMSR (A), MPD (B), R50 (C), and R90 (D). Spearman correlation coefficients (*r*) and corresponding *p* values are shown in each panel. Annualized changes in the metrics are reported in pixels per year (where 1 pixel corresponds to a physical dimension of 0.15 mm).

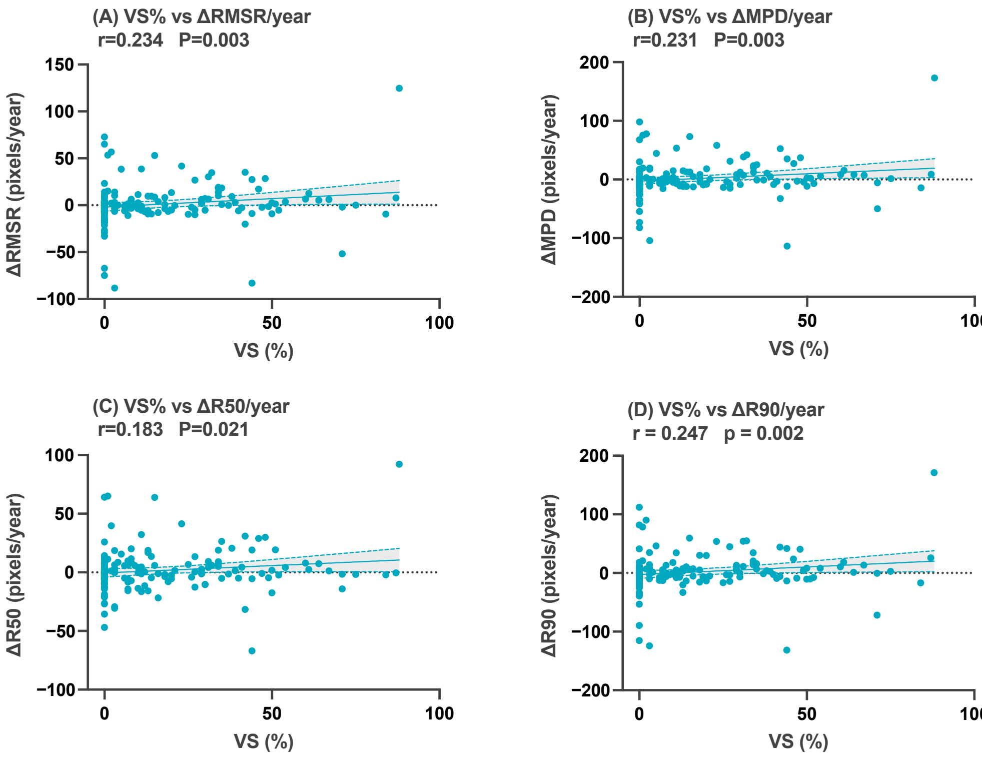

Figure S7. Correlations between BL VS% and annualized changes in AI-derived compactness metrics. Scatter plots showing the relationships between BL VS% and annualized changes in RMSR (A), MPD (B), R50 (C), and R90 (D). Spearman correlation coefficients (*r*) and corresponding *p* values are shown in each panel. Annualized changes in the metrics are reported in pixels per year (where 1 pixel corresponds to a physical dimension of 0.15 mm).

Supplementary Tables

Table S1. Sensitivity analyses of the associations between baseline ultrasound inflammatory metrics and annualized changes in AI-derived compactness metrics after additional adjustment for baseline DAS28-ESR.

BL ultrasound metric	Outcome	β	95% CI	Raw p	FDR-adjusted q	R^2
GS	ΔRMSR/year	1.832	−0.170 to 3.833	0.0725	0.1784	0.2863
GS	ΔMPD/year	2.579	−0.098 to 5.257	0.0589	0.1784	0.2450
GS	ΔR50/year	1.858	0.125 to 3.591	0.0358	0.1784	0.1414
GS	ΔR90/year	2.821	0.006 to 5.637	0.0496	0.1784	0.3165
PD	ΔRMSR/year	1.235	−0.656 to 3.127	0.1989	0.2114	0.2788
PD	ΔMPD/year	1.777	−0.755 to 4.309	0.1677	0.1929	0.2366
PD	ΔR50/year	1.259	−0.381 to 2.900	0.1313	0.1784	0.1292
PD	ΔR90/year	2.048	−0.610 to 4.706	0.1300	0.1784	0.3094
GS+PD	ΔRMSR/year	0.785	−0.204 to 1.774	0.1191	0.1784	0.2825
GS+PD	ΔMPD/year	1.116	−0.208 to 2.440	0.0980	0.1784	0.2408
GS+PD	ΔR50/year	0.798	−0.059 to 1.655	0.0679	0.1784	0.1353
GS+PD	ΔR90/year	1.249	−0.142 to 2.639	0.0781	0.1784	0.3131
VS%	ΔRMSR/year	0.104	−0.060 to 0.269	0.2114	0.2114	0.2784
VS%	ΔMPD/year	0.154	−0.066 to 0.374	0.1688	0.1929	0.2366
VS%	ΔR50/year	0.111	−0.031 to 0.253	0.1244	0.1784	0.1297
VS%	ΔR90/year	0.176	−0.055 to 0.407	0.1338	0.1784	0.3092

Each model was adjusted for the corresponding BL compactness metric, BL wrist radiographic score, age, sex, and BL DAS28-ESR. Raw p values for the associations between BL ultrasound inflammatory metrics and the four annualized compactness outcomes were adjusted using the Benjamini–Hochberg false discovery rate procedure as one family of 16 tests. FDR-adjusted q values were calculated using the unrounded raw p values, and $q < 0.05$ was considered statistically significant. Bold values indicate nominal statistical significance based on raw $p < 0.05$. None of the associations remained statistically significant after FDR correction.

Table S2. Sensitivity analyses of the associations between baseline ultrasound inflammatory metrics and annualized changes in AI-derived compactness metrics after additional adjustment for baseline DAS28-CRP.

BL ultrasound metric	Outcome	β	95% CI	Raw p	FDR-adjusted q	R^2
GS	ΔRMSR/year	1.853	−0.146 to 3.852	0.0690	0.1737	0.2857
GS	ΔMPD/year	2.619	−0.056 to 5.295	0.0549	0.1737	0.2440
GS	ΔR50/year	1.868	0.137 to 3.598	0.0346	0.1737	0.1413
GS	ΔR90/year	2.832	0.021 to 5.643	0.0483	0.1737	0.3165
PD	ΔRMSR/year	1.257	−0.632 to 3.146	0.1905	0.2029	0.2782
PD	ΔMPD/year	1.818	−0.712 to 4.347	0.1577	0.1823	0.2355
PD	ΔR50/year	1.270	−0.367 to 2.907	0.1273	0.1737	0.1291
PD	ΔR90/year	2.061	−0.592 to 4.714	0.1268	0.1737	0.3094
GS+PD	ΔRMSR/year	0.796	−0.192 to 1.784	0.1135	0.1737	0.2819
GS+PD	ΔMPD/year	1.136	−0.186 to 2.459	0.0916	0.1737	0.2398
GS+PD	ΔR50/year	0.803	−0.053 to 1.659	0.0656	0.1737	0.1352
GS+PD	ΔR90/year	1.255	−0.134 to 2.643	0.0761	0.1737	0.3131
VS%	ΔRMSR/year	0.106	−0.058 to 0.270	0.2029	0.2029	0.2777
VS%	ΔMPD/year	0.157	−0.062 to 0.377	0.1595	0.1823	0.2355
VS%	ΔR50/year	0.112	−0.030 to 0.254	0.1209	0.1737	0.1295
VS%	ΔR90/year	0.177	−0.053 to 0.407	0.1303	0.1737	0.3092

Each model was adjusted for the corresponding BL compactness metric, BL wrist radiographic score, age, sex, and BL DAS28-CRP. Raw p values for the associations between BL ultrasound inflammatory metrics and the four annualized compactness outcomes were adjusted using the Benjamini–Hochberg false discovery rate procedure as one family of 16 tests. FDR-adjusted q values were calculated using the unrounded raw p values, and $q < 0.05$ was considered statistically significant. Bold values indicate nominal statistical significance based on raw $p < 0.05$. None of the associations remained statistically significant after FDR correction.
